# Antimicrobial Activity of Gallium(III) Compounds: Pathogen-Dependent Targeting of Multiple Iron/Heme-Dependent Biological Processes

**DOI:** 10.3390/cimb46080541

**Published:** 2024-08-22

**Authors:** Seoung-ryoung Choi, Mohammed A. Hassan, Bradley E. Britigan, Prabagaran Narayanasamy

**Affiliations:** 1 Department of Pathology, Microbiology and Immunology, College of Medicine, University of Nebraska Medical Center, Omaha, NE 68198, USA; 2 Department of Internal Medicine, College of Medicine, University of Nebraska Medical Center, Omaha, NE 68198, USA

**Keywords:** gallium, antimicrobial activity, mechanisms of action, reactive oxygen species

## Abstract

Metals play vital roles in biological systems, with iron/heme being essential for cellular and metabolic functions necessary for survival and/or virulence in many bacterial pathogens. Given the rise of bacterial resistance to current antibiotics, there is an urgent need for the development of non-toxic and novel antibiotics that do not contribute to resistance to other antibiotics. Gallium, which mimics iron, has emerged as a promising antimicrobial agent, offering a novel approach to combat bacterial infections. Gallium does not have any known functions in biological systems. Gallium exerts its effects primarily by replacing iron in redox enzymes, effectively inhibiting bacterial growth by targeting multiple iron/heme-dependent biological processes and suppressing the development of drug resistance. The aim of this review is to highlight recent findings on the mechanisms of action of gallium and provide further insights into the development of gallium-based compounds. Understanding the mechanisms underlying gallium’s biological activities is crucial for designing drugs that enhance their therapeutic therapies while minimizing side effects, offering promising avenues for the treatment of infectious diseases.

## 1. Introduction

The discovery of antibiotics has had a huge impact on humanity, saving countless lives from bacterial infections. However, overuse and misuse have led to the development of bacterial resistance to drugs, resulting in increased fatalities. The rise of drug-resistant bacteria has posed a significant challenge to treating bacterial infections, highlighting the need for the development of new antibiotics that do not promote bacterial resistance.

The use of metals in medicine dates back many centuries. Although essential metals can be toxic to cells in high concentrations, non-essential metals like silver (Ag), mercury (Hg), gold (Au), titanium (Ti), and tellurium (Te) are highly toxic to most bacteria and possess potent microbicidal properties, even at very low concentrations [1,2,3]. Over the past two hundred years, medical practitioners have employed oxides of Te, Mg, and As, as well as salts of Cu and Hg, to treat various diseases such as leprosy, tuberculosis, gonorrhea, and syphilis [2]. The practice of using metals in medicine was common until Nobel laureate Sir Alexander Fleming discovered antibiotics in the 1920s, which led to a rapid decline in these applications. However, with the increasing prevalence of bacterial resistance to existing antibiotics, there is a critical need to develop novel, non-toxic antibiotics that do not contribute to resistance against other antibiotics. Consequently, metallodrugs have gained attention for combating drug-resistant bacteria. Among these, gallium [Ga(III)], a semi-metallic element, is utilized in various applications and devices, including electronics, semiconductors, and medical imaging. Ga(III) has shown therapeutic effects in the treatment of various disorders, including cancer, hemostasis, bone-related diseases, and autoimmune diseases [4]. Gallium nitrate (Ga(NO_3_)_3_) in citrate buffer was approved by the Food and Drug Administration (FDA) for the treatment of hypercalcemia secondary to malignancy in 2003.

Remarkably, Ga(III) also exhibits broad-spectrum antimicrobial activity against many pathogens. Gallium shares several chemical and physical properties similar to iron, such as ionization potential, atomic radius, and electron configuration. In octahedral co-ordination geometry, the ionic radius of Ga^3+^ is 0.62 Å, while that of Fe^3+^ (high spin) is 0.645 Å [5]. The tetrahedral ionic radius of Ga^3+^ and Fe^3+^ are 0.47 Å and 0.49 Å, respectively [6]. These properties enable Ga(III) to mimic Fe(III), allowing it to bind to the binding sites on proteins where Fe(III) binds. However, unlike iron, Ga(III) does not undergo redox recycling, whereas Fe(III) is readily reduced to Fe(II) under physiological conditions. Ga(III) has shown antimicrobial activity against various bacterial pathogens because microorganisms cannot distinguish between the two metals. Enzymes substituted with Ga(III) cannot perform essential functions necessary for bacterial survival, resulting in the disruption of iron-dependent metabolic processes, including iron acquisition/utilization, electron transport, oxidative stress defense, and DNA synthesis [6].

Other antimicrobial metals, such as Ag and Cu, exhibit antimicrobial activity by reacting with reactive functional groups or by generating reactive oxygen species (ROS) from reactions with water and air. These mechanisms have led to the development of bacterial resistance through an efflux pump, conversion of the toxic metal to a less toxic form by modification, or sequestration by a metal-binding protein or ligands [2]. In contrast, Ga(III) exerts its antimicrobial activity by mimicking Fe(III), an essential element for bacterial survival. This mechanism likely makes it much more difficult for bacteria to develop resistance to gallium, as reducing Ga(III) uptake would lead to the reduction of iron uptake, which is vital for bacterial growth [7,8].

Due to multi-targeting antimicrobial activity against bacteria, including drug-resistant strains, Ga(III) has garnered significant attention as a novel antibacterial strategy. Consequently, Ga(III)-based compounds, including combination therapy using different forms of Ga(III), have been developed in a variety of ways to increase therapeutic efficacy and improve their low bioavailability. Ga(III) is hydrolyzed into various hydroxide species, such as insoluble Ga(OH)_3_ and soluble Ga(OH)_4_, under physiological conditions [9]. The formation of Ga(III) complexes is one way to improve solubility and protect Ga(III) from hydrolysis, thereby increasing its bioavailability. For example, gallium nitrate (Ganite^TM^), which is no longer available for clinical use in the US for reasons other than safety or effectiveness, needs to be administrated intravenously due to hydrolysis [10].

The usefulness of an antibiotic is increased if it has good solubility and bioavailability when administered orally. To increase gallium’s oral bioavailability, gallium-based compounds have been prepared by complexing Ga(III) ions with various ligands [11]. Gallium maltolate (GaM), composed of three maltolate ligands bidentately bound to a gallium ion, exhibits some activity as both an anticancer and antimicrobial agent following oral administration [12]. Researchers continue to explore its therapeutic applications, and GaM is still under investigation for the treatment of relapsed and refractory glioblastoma. Another oral gallium complex, tris(8-quinolinolato)gallium(III) (KP46), was synthesized for anticancer therapy and showed high hydrolytic stability [12,13,14]. Although KP46 exerted promising activity against renal cancer and melanoma cells [15], it has poor bioavailability, possibly due to its low water solubility [13]. Duffin et al. also synthesized alkyl gallium quinolinolate complexes, which exhibited good activity against the motile promastigote form of *Leishmania major* with micromolar ranges of IC_50_ [16].

These gallium complexes demonstrate increased stability, solubility, and bioavailability, effectively delivering the antimicrobial agent Ga(III) to target sites and enhancing its therapeutic potential. These complexes also reduce potential side effects associated with free Ga(III) ions. To further improve the efficacy and safety of Ga(III)-based antimicrobial therapies, researchers are investigating various ligands for Ga(III) complexation. They are also developing innovative delivery systems, such as nanomaterials, gallium alloys, and liquid metals for controlled release and targeted delivery of Ga(III) [7,17,18].

This review summarizes the mechanisms of action of Ga(III) to offer insights into enhancing the antibacterial activity of Ga(III)-based compounds. Understanding the mechanisms underlying gallium’s biological activities will enhance its therapeutic therapies for the treatment of infectious diseases.

## 2. Mechanisms of Action of Ga(III)-Based Compounds

### 2.1. Iron Acquisition Pathways as a Target for Gallium Antimicrobials

**Figure 1:**
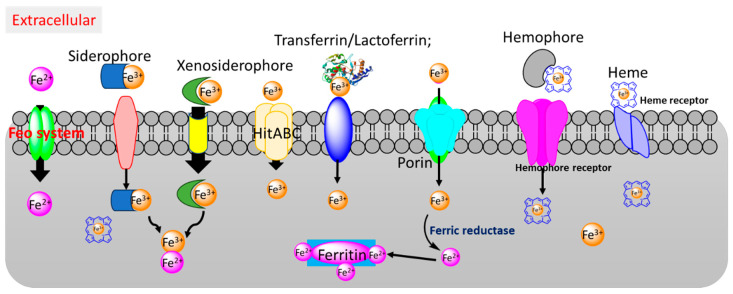
Bacterial iron/heme uptake pathways. Feo system: bacterial ferrous iron transport. HitABC: a ferric iron ABC transport system. Ferritin: there are two types of bacterial iron storage proteins, bacterial ferritin and bacterioferritin.

Iron plays multiple roles in biological processes, including DNA synthesis, electron transport, catalysis, oxygen transport, and cellular respiration [19]. Bacteria have evolved numerous strategies to acquire iron from their environment, including animal hosts, to ensure their survival and proliferation [20]. Bacteria possess various iron-uptake mechanisms to compete with host cells and systems for iron, addressing the challenges posed by the low bioavailability of iron and the host’s nutritional immunity, the latter referring to biological processes designed to sequester iron from invading microbes. These bacterial iron uptake mechanisms include siderophore/hemophore-mediated uptake, ferrous iron transporters (Feo system), ferric iron transporters, and heme receptors (Figure 1).

Pathogenic bacteria upregulate siderophore biosynthesis and iron-trafficking pathways in iron-deficient environments. Bacterial siderophores, ferric iron (Fe^3+^) chelators, sequester and deliver Fe^3+^ into bacteria from the environment [21,22]. These siderophores can compete with and, in some cases, remove Fe^3+^ from lactoferrin, transferrin, and other iron-binding proteins due to their higher affinity for Fe^3+^ [23,24,25]. Researchers have exploited siderophores to deliver therapeutic agents into pathogens, including Ga(III) ions and antibiotics. A siderophore desferrioxamine (DFO), produced by *Streptomyces pilosus*, was used to deliver Ga(III) to bacteria. The Ga(III)-DFO complex demonstrated bactericidal activity against *P. aeruginosa* and inhibited biofilm formation [26]. Similarly, Ga(III)-loaded pyochelin, a siderophore from *P. aeruginosa*, significantly enhanced the inhibitory effect on *P. aeruginosa* growth compared to gallium nitrate [27]. Pandy. et al. synthesized and demonstrated that a Ga(III)-ciprofloxacin-functionalized desferrichrome complex (gallium-sideromycin conjugate) exhibits broad-spectrum inhibitory activity against Gram-negative and -positive bacteria [28]. This complex is internalized via *FuhA*-mediated transport. Thus, siderophore-mediated drug delivery is a promising strategy for enhancing antibacterial effects by increasing the uptake and activity of the drug.

Like Gram-positive and Gram-negative bacteria, iron acquisition by *M. tuberculosis* involves both siderophore-mediated iron acquisition and heme-iron acquisition. *M. tuberculosis* relies on siderophores (exochelins, carboxymycobactin, and mycobactin) to obtain iron from its environment for its growth and virulence [29,30,31]. Thus, targeting siderophore biosynthesis pathways is a rational approach to interrupting siderophore-mediated iron acquisition. Many studies provide detailed research on developing inhibitors for these pathways, particularly the mycobactin biosynthesis pathway [32,33,34]. As mentioned above, another approach for developing antitubercular drugs is to use siderophore as a vehicle to enhance the uptake of a compound with antimicrobial activity. A sideromycin, mycobactin-artemisinin conjugate, shows inhibitory activity against multi-drug-resistant and extremely drug-resistant strains of *M. tuberculosis* and malaria [35].

Currently, no studies on complexes of Ga(III) with siderophores or Ga(III)-sideromycins from *M. tuberculosis* as antitubercular agents have been reported. However, Ga-transferrin demonstrated a Fe-reversible, concentration-dependent growth inhibition of *M. tuberculosis* strains and *M. avium* complex (MAC) when the organisms grew extracellularly or within human macrophages [36].

### 2.2. Heme Acquisition Pathways as a Target for Antimicrobials

In the human host, the most abundant source of iron is heme, a complex of iron with protoporphyrin. Heme is an important prosthetic group of heme proteins, such as hemoglobin, myoglobin, and other heme-containing enzymes responsible for oxygen transport, electron transfer, and redox reactions. Most heme is found in hemeproteins, including hemoglobin in erythrocytes and myoglobin in muscle tissue [20,37]. These hemeproteins and free heme are primary targets for bacterial pathogens to obtain iron.

While siderophores recognize and bind ferric ions in the environment, they cannot remove iron from the heme. Consequently, many bacteria secrete hemophores to capture heme and heme analogs (Figure 1). The heme can be either used directly to meet the metabolic needs of the organism for heme or broken down, thereby releasing iron, which is then available for use. The mechanisms for sequestering free heme or heme within hemeproteins and transporting them are diverse among bacterial species [38]. Several reviews have been reported describing heme-uptake mechanisms in Gram-positive and Gram-negative bacteria, as well as mycobacteria [21,38,39,40,41]. Here, we provide a brief overview of heme uptake by *M. tuberculosis*. In *M. tuberculosis*, several genes have been identified that are involved in heme uptake, allowing the mycobacterium to use heme as an alternative iron source. Proteins like Rv0203, mmpL11, and mhuD are implicated in heme utilization. Rv0203 is a secreted heme-binding protein, but it is not able to transfer heme from hemoglobin to inner membrane proteins such as MmpL3 and MmpL11 due to its low heme affinity compared to hemophore HasA from *P. aeruginosa* [42,43].

Interestingly, it was reported that proline-proline-glutamate (PPE) proteins characterized in *M. tuberculosis* are involved in heme uptake. The PPE proteins are unique to mycobacteria. Tullius et al. found that PPE37 is an essential protein for efficient heme uptake as an iron source by *M. tuberculosis* [44], while Mitra et al. identified novel heme-binding proteins, including PPE36, PPE62, and FecB2 (Rv0265c), with distinct roles in heme utilization [45]. PPE36 and PPE62 act as cell surface receptors for heme. The deletion of the *ppe36* gene of *M. tuberculosis* significantly reduced heme utilization, suggesting that PPE36 is crucial for efficient heme utilization in vitro [45]. It has been suggested that the periplasmic protein FecB2 possibly plays a role in both ferric siderophore and heme acquisition pathways. However, these proteins have low heme-binding affinity in bacterial heme uptake pathways compared to other proteins, such as IsdC and HasA [46,47].

Due to their high structural homology to heme, these heme-uptake pathways have inspired the development of non-iron metalloporphyrins as antimicrobials [11,48]. These metalloporphyrins target either hemeproteins or pathways involved in heme uptake. Many non-iron metalloporphyrins, which mimic heme, were tested against the sexually transmitted pathogens (*Neisseria gonorrhoeae* and *Haemophilus ducreyi*), with gallium protoporphyrin IX (GaPP) showing the highest activity without causing the major side effect in vivo [49]. Likewise, GaPP inhibits the growth of *P. aeruginosa* by binding cytochrome in a place of heme, disrupting electron transfer during oxidative phosphorylation [50]. Recently, Zhang et al. demonstrated that water-soluble cationic gallium protoporphyrin (Ga-CHP) exerts broad-spectrum antibacterial activity against both Gram-positive and Gram-negative bacteria, including multi-drug-resistant strains [51]. Additionally, Ga-CHP acts as a photosensitizer, enhancing its antibacterial efficacy through synergistic photodynamic therapy in vitro and in vivo.

Similar to siderophore–antibiotic conjugates, non-iron metalloporphyrins can be linked with antibiotics to enhance the delivery of drugs across the membrane, thereby increasing the permeability of the lipid membrane for a specific antibiotic. While no metalloporphyrin–antibiotic conjugates have been identified that exploit the bacterial heme-uptake pathways for significantly enhanced dual actions, porphyrin–antibiotic conjugates have attracted attention for their use in antibacterial photodynamic therapy (PDT). One research group prepared a 5,10,15,20-tetrakis (para-aminophenyl) porphyrin–vancomycin conjugate and demonstrated that the conjugate as a photosensitizer exerts effective and selective inhibitory activity against *Staphylococcus aureus* over *Escherichia coli* under white light [52]. A conjugate of 5(4′-carboxyphenyl)-10,15,20-triphenylporphyrin (cTPP) with the cationic antimicrobial peptide apidaecin exhibited broad-spectrum antibacterial activity when activated by light, effectively inhibiting bacterial growth, including *P. aeruginosa* and *S. aureus* [53].

### 2.3. Potential Targets for Ga(III)-Based Compounds

#### 2.3.1. Ribonucleotide Reductase (RNR)

One strategy for developing new antibacterial drugs focuses on finding more selective compounds that target multiple pathways to minimize drug resistance. RNR catalyzes the transformation of ribonucleotides to deoxyribonucleotides, which are necessary for DNA synthesis and essential for cell proliferation, via a mechanism involving protein radicals [54,55]. RNR consists of two subunits, α (or R1) and β (or R2). The R2 subunit contains an indispensable binuclear iron center, where Ga(III) ion replaces iron, thereby disrupting the catalysis. There are several studies supporting the enzymatic inhibition by Ga(III). A molecular modeling study revealed that the redox-inactive Ga(III) replaces the redox-active Fe(III), leading to the inhibition of the catalysis [56]. In addition to the substitution of Fe(III) in the R2 subunit, it has been suggested that gallium nitrate can inhibit enzyme activity by forming Ga(III)-nucleotide diphosphate (NDP) complexes [57,58,59]. A density functional theory (DFT)/polarizable continuum model (PCM) study revealed that Ga(III) ion forms complexes with the free NDPs, which are not/poorly recognizable by the RNR, thus inhibiting the RNR activity in malignant cells [58]. Overall, it appears that Ga(III) inhibits RNR activity via several mechanisms, including iron deprivation and the formation of unfavorable Ga(III)-NDP complexes as substrates to RNR.

**Figure 2:**
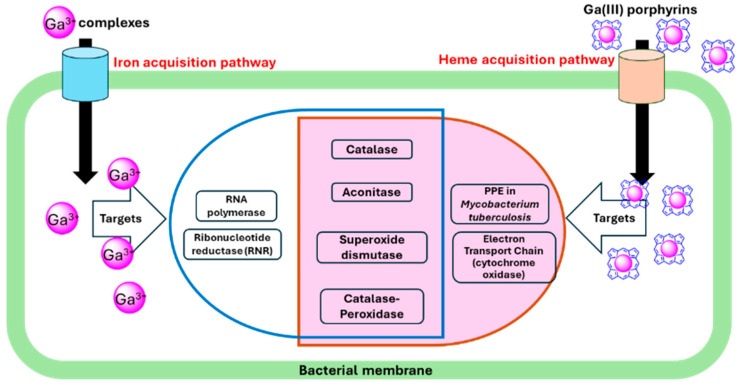
Schematic diagram of the uptake of gallium-based compounds through iron/heme acquisition pathways and their proposed intracellular targets.

Gallium nitrate showed promising results when used in mice infected with *M. tuberculosis* [60]. This study demonstrated that the Ga(III) antimycobacterial activity paralleled its ability to inhibit several enzymes, including RNR and aconitase (Figure 2). In addition to bacteria, gallium nitrate inhibits eukaryotic cell proliferation by inhibiting RNR activity, accounting in part for its efficacy as an anticancer drug [61]. Consistent with the result observed in *M. tuberculosis*, gallium nitrate has been shown to inhibit *P. aeruginosa* RNR by up to ~40% at concentrations that result in growth inhibition [8]. The authors noted that increasing the gallium concentration did not lead to further inhibition, suggesting that gallium may specifically inhibit one of the two classes of *P. aeruginosa* RNR [62].

#### 2.3.2. Oxidative Phosphorylation and Cytochrome Oxidases

The heme mimetic GaPP has been shown to possess broad antibacterial activity [11,63]. Stojiljkovic et al. were the first to demonstrate that non-iron metalloporphyrin compounds possess such activity, with GaPP being the most effective [48]. The authors proposed that bacteria internalize the metalloporphyrin via heme-uptake pathways. Once inside the cell, the metalloporphyrin binds to cytochromes, disrupting the electron transfer in oxidative phosphorylation (Figure 2). This disruption causes incomplete O_2_ reduction and generation of ROS [48]. Arrest of cellular respiration was also observed in *P. aeruginosa*, where GaPP inhibits the aerobic growth of *P. aeruginosa* by targeting cytochrome oxidases [50]. The authors investigated all five terminal oxidases of *P. aeruginosa* to determine which ones were responsible for GaPP sensitivity, as they require heme as a cofactor. Their findings indicated that the Cco-1, Cco-2, and Cio terminal oxidases were the primary targets for GaPP.

#### 2.3.3. RNA Polymerase

Iron-targeting gallium compounds demonstrate antimicrobial activity through multiple mechanisms of action that remain unclear. The combined study of metalloproteomics with metabolomics and transcriptomics revealed that gallium nitrate binds RpoB and RpoC, two subunits of RNA polymerase in *P. aeruginosa*, leading to the reduction of metabolic rates and energy utilization by suppressing RNA synthesis [64]. Furthermore, the combination of gallium nitrate with acetate was found to be synergistic in inhibiting the growth of persister cells and was efficacious in murine *P. aeruginosa* infection models, attenuating bacterial virulence.

#### 2.3.4. Aconitase

Several studies imply that Ga(III)-exposed bacteria exhibit reduced activity of multiple iron-utilizing enzymes, including aconitase, catalase, and iron superoxide dismutase. Aconitase is a Fe-sulfur enzyme that catalyzes the isomerization of citrate to isocitrate via cis-aconitate in the tricarboxylic acid cycle. The activity of aconitase from *M. tuberculosis* is reduced when exposed to gallium nitrate [60]. Likewise, an inhibitory effect of gallium on aconitase was observed in *M. abscessus*, with both gallium nitrate and GaPP inhibiting its aconitase activity [65]. The authors proposed a plausible explanation that Ga(III) ion, released from the breakdown of GaPP by bacterial heme oxygenase, may prevent the formation of the iron-sulfur cluster essential for aconitase catalysis. An alternative explanation for the reduced aconitase activity is that ROS generated by Ga(III) compounds, including GaPP, might disrupt the enzyme’s iron-sulfur cluster (see discussion below). Also, synergistic inhibition of aconitase was observed when *M. abscessus* was exposed to a combination of gallium nitrate and GaPP [65].

However, the reduction of aconitase activity by Ga(III) seems to be pathogen-dependent. Gallium nitrate did not have a significant impact on *P. aeruginosa* aconitase activity [8]. The inhibition of aconitase activity by Ga(III) may vary due to the differences in gene regulation associated with the inhibitory activity of Ga(III) compounds between mycobacteria and Gram-negative bacteria. Thus, further research is needed to provide insight into inhibiting aconitase activity by Ga(III) in these bacterial types, including Gram-positive bacteria.

#### 2.3.5. Oxidative Stress and Antioxidant Enzymes

**Figure 3:**
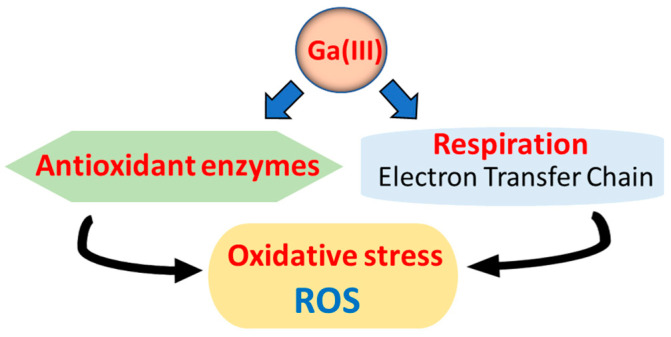
Reactive oxygen species (ROS) induced by Ga(III) in bacteria. Ga(III) compounds can inhibit antioxidant enzymes, accumulating ROS within bacteria. In addition, Ga(III) porphyrin, such as GaPP, disrupts the electron transfer chain (ETS) by replacing heme in respiratory enzymes, such as cytochrome c. This leads to incomplete oxygen reduction, generating superoxide and other ROS.

Antioxidant enzymes play essential roles in protecting the cells from oxidative stress caused by ROS that damage cellular components such as DNA, proteins, and lipids. Bacteria have evolved various antioxidant enzymes, including catalases, superoxide dismutases, and peroxidases to defend the cells from these ROS. Recent studies have shown that Ga(III) leads to ROS accumulation through two mechanisms: direct inhibition of antioxidant enzymes or indirect promotion of ROS production (Figure 3). Zemke et al. demonstrated that ROS might play a role in the antibacterial mechanisms of Ga(III) by showing that gallium nitrate exhibits a better antibacterial effect on aerobic bacteria compared to anaerobic bacteria [66]. They also demonstrated the synergistic effect of gallium nitrate and sodium nitrite as a NO donor in inhibiting *P. aeruginosa* growth and preventing biofilm growth under both aerobic and anaerobic conditions, indicating the involvement of ROS induced by gallium nitrate. Another study with *P. fluorescens* supported the finding that Ga(III) disrupts Fe metabolism and induces oxidative stress that a reductive activity can neutralize via the overexpression of NADPH-producing enzymes [67].

However, intracellular ROS levels measured by the ROS fluorescent probe H2DCFDA indicated that gallium nitrate did not significantly affect ROS levels in *P. aeruginosa* [68,69]. Despite this, gallium nitrate exhibits bacteriostatic growth-inhibitory activity against *P. aeruginosa*, which was accompanied by decreased ATP production [69]. Thus, further studies are required to identify the cellular targets for Ga(III) ion and provide new insights into its mechanism of antibacterial action.

#### 2.3.6. Catalase

The antibacterial effects of gallium compounds, such as Ga(III) ions or Ga(III) porphyrins, have been suggested to be partly due to the inhibition of bacterial antioxidant enzymes, resulting in an accumulation of ROS. Catalase is a key antioxidant found in many organisms, including bacteria. It converts hydrogen peroxide (H_2_O_2_) into water and oxygen [70,71]. Catalase is a tetrameric enzyme containing four Fe(II)-bound hemes, which is essential for its catalytic activity. Thus, catalase is a potential target for non-iron metalloporphyrins, including Ga(III) porphyrins, resulting in the disruption of antioxidant activity [48,65].

Brugna et al. developed an excellent bacterial system to determine the mechanism of antibacterial non-iron metalloporphyrins [72]. The Gram-positive bacteria *Enterococcus faecalis* does not depend on heme for growth, leading to resistance to non-iron metalloporphyrins. The authors exploited this feature for the production of non-iron metalloporphyrin-substituted catalases. They found that catalases containing non-metalloporphyrins, especially GaPP, showed significantly reduced activity compared to heme-substituted catalase. In addition, an H_2_O_2_ consumption assay and native gel studies confirmed that GaPP inhibits catalase in *S. aureus*, *P. aeruginosa*, and *M. abscessus* [65,68,73]. These results suggest that GaPP binds to apo-catalase, rendering it inactive in the decomposition of H_2_O_2_.

A contradictory observation has been reported regarding the effect of gallium nitrate on catalase activities. Gallium nitrate exhibited pathogen/strain-dependent inhibitory activity on catalase. Gallium nitrate, which is not a cofactor for catalase, reduced catalase activity in *P. aeruginosa* PAO1 and *Francisella novicida* [8,74,75], while no inhibition was observed in *Staphylococcus aureus*, *P. aeruginosa* PA103, and *M. abscessus* [65,68,73]. In the case of *P. aeruginosa* strains, the authors suggested that this discrepancy might be due to varying iron concentration in the media used to grow the bacteria, indicating further study is needed to explore strain-specific or growth medium-related differences in antimicrobial activity of Ga(III) ion [68]. However, those catalase activity assays, including H_2_O_2_ consumption and native gel assays, were performed using lysates from Ga(III)-treated bacteria, whose measurements might have been affected by other chemical substances or enzymes in the lysates, resulting in inaccurate results. Guo et al. reported more convincing mechanistic studies by performing RNA sequencing (RNA-seq) [76]. They demonstrated that the transcriptional levels of two superoxide dismutase-encoding genes, sodB and sodC, and two catalase-encoding genes, katE and katG, were downregulated by gallium nitrate, leading to the intracellular accumulation of ROS in *K. pneumoniae*. Thus, it appears that gallium nitrate may alter transcriptional gene expressions rather than directly inhibiting catalase.

#### 2.3.7. Superoxide Dismutase

The superoxide dismutases are additional key bacterial antioxidant enzymes [77,78]. Superoxide dismutases (SOD) are metalloenzymes that catalyze the dismutation of superoxide to H_2_O_2_ and O_2_ using a redox-active metal. To date, four isoforms of SODs have been identified: MnSOD, FeSOD, Cu/ZnSOD, and NiSOD [79,80]. MnSOD and FeSOD are typically located in the cytosol, while Cu/ZnSOD is found in periplasmic space. Since Ga(III) ion can substitute for Fe(III) in many redox enzymes, it is thought that Ga(III) may interfere with bacterial SODs by replacing a metal, most likely iron, in the SOD, leading to ROS accumulation within bacteria.

The SODs of *P. aeruginosa* and *K. pneumoniae* have been investigated as potential targets for GaPP and gallium nitrate [8,68,74]. Similar to catalase, these studies yielded inconsistent results: Gallium nitrate inhibited SOD activity from *P. aeruginosa* PA103 but did not from *P. aeruginosa* PAO1 and *K. pneumoniae*. In addition, a study with Ga(III)-chelated transferrin or lactoferrin reduced FeSOD activity in *F. novicida* [75]. The inconsistencies could be attributed to differences in iron concentrations in the growth media, pathogen-specific variations, or interference from the assays using bacterial lysates. Thus, SOD, as one of the potential targets for Ga(III), needs to be evaluated by performing alternative assays, such as RNA sequencing and proteomics.

#### 2.3.8. Peroxidases

Bacterial peroxidases play a crucial role in protecting cells from oxidative damage. These enzymes convert H_2_O_2_ and organic peroxides into water and alcohols, respectively. Catalase-peroxidase (KatG) and alkyl hydroperoxide reductase (ahpC) limit oxidative stress in *M. tuberculosis*. KatG is a bifunctional heme-containing enzyme that protects *M. tuberculosis* from H_2_O_2_. Notably, unlike other mycobacteria, *M. tuberculosis* relies solely on KatG for H_2_O_2_ detoxification. In addition, KatG also possesses peroxynitritase activity [81], indicating that KatG is an important virulence factor. Given this unique dependency on KatG for protection, Ga(III) porphyrins, acting as a heme mimic, could serve as potential antitubercular agents against *M. tuberculosis* by targeting this enzyme. However, the effectiveness and mechanisms of Ga(III) porphyrins as antitubercular agents, particularly in vivo, remain to be fully elucidated.

Interestingly, gallium nitrate enhanced the iron-containing peroxidase activity of *Chrorella pyrenoidosa* in both iron-deficient and normal iron-supplied cultures [82]. Similarly, the effects of gallium curcumin and gallium diacetylcurcumin on the structure, function, and oxidative stability of the horseradish peroxidase (HRP) enzyme were evaluated [83]. These gallium complexes increased the antioxidant activity of the peroxidase enzyme, suggesting that the complexes exhibit the potential for cancer treatment, but they have no significant antibacterial activity.

## 3. Conclusions and Prospective Views

Iron is an essential element for pathogenic bacterial survival in human hosts, playing a critical role in multiple biological processes. Due to their similar physicochemical properties to iron, gallium-based compounds have emerged as potential antimicrobial agents. Thus, gallium, acting as a multi-target antimicrobial agent, has been demonstrated to exert a broad antibacterial activity by interrupting iron/heme metabolisms. Notably, it shows inhibitory activity against pathogenic bacteria that are resistant to current antibiotics. Numerous enzymes have been identified as targets for Ga(III) ions and Ga(III) porphyrins. One compelling mechanism of action for Ga(III)-based compounds is their ability to increase the production of ROS in bacteria by disrupting enzymatic antioxidants, such as SODs, catalases, peroxidases, or non-enzymatic antioxidant processes. Thus, Ga(III) enhances the host’s oxidative defenses during the immune response to pathogenic infection. However, it appears that Ga(III)’s inhibitory activity and mechanism(s) of action are pathogen-dependent (Table 1). For example, *E. coli* SOD and catalase mutants exert hypersensitivity to GaPP due to the overproduction of ROS [48]. In contrast, MRSA catalase and SOD transposants do not show increased susceptibility to GaPP and gallium nitrate [73]. Furthermore, MRSA transposant mutants with defects in antioxidant activity, such as thiol peroxidase, alkyl hydroperoxide reductase (AhpC), and staphyloxanthin, exhibit susceptibility to these gallium compounds comparable to that of MRSA USA300. These findings suggest that Ga(III)’s antimicrobial activity involves targeting multiple iron/heme-dependent biological processes and that ROS produced by Ga(III) is not the only factor contributing to bacterial killing. While gallium shows promise as an antimicrobial agent by interrupting iron/heme metabolism, elucidation of the mechanism of the antibacterial activity of gallium-based compounds would improve their efficacy as antibiotics.

Finally, in addition to chelating various organic ligands with Ga(III) to form complexes, various technologies have been utilized to enhance the antimicrobial activity of Ga(III). Ga(III)-loaded nanomaterials, including nanoparticles and Ga(III)-doped alloys, are of interest. Developing nanomaterials that enable sustained and controlled Ga(III) delivery could significantly enhance the antibacterial activity and advance the development of antibacterial agents.

## Figures and Tables

**Table 1:** Gallium(III) compounds and their pathogen-specific targets.

Ga(III)	Target	Bacteria	References
Ga(NO_3_)_3_	Aconitase	*M. tuberculosis*, *M. abscessus*	[60,65]
	Ribonucleotide reductase	*M. tuberculosis*, *P. aeruginosa* PAO1	[8,60]
	Catalase	*M. abscessus*, *P. aeruginosa* PAO1, *K.pneumoniae*, *F. novicida*	[8,68,74,75]
	Superoxide dismutase	*P. aeruginosa* 103	[68]
GaPP	Catalase	MRSA, *K. pneumoniae*, *P. aeruginosa* 103, *M. abscessus*	[65,68,73,74]
	Superoxide dismutase	*P. aeruginosa* 103	[68]
	Aconitase	*M. abscessus*	[65]

GaPP: Ga(III) protoporphyrin IX.
